# Inhibition of STAT1 sensitizes radioresistant nasopharyngeal carcinoma cell line CNE-2R to radiotherapy

**DOI:** 10.18632/oncotarget.19690

**Published:** 2017-07-29

**Authors:** Song Qu, Ya Guo, Shi-Ting Huang, Xiao-Dong Zhu

**Affiliations:** ^1^ Department of Radiation Oncology, The Affiliated Tumor Hospital of Guangxi Medical University, Cancer Institute of Guangxi Zhuang Autonomous Region, Key Laboratory of High Incidence Tumor Prevention and Treatment Guangxi Medical University, Ministry of Education, Nanning, 530021, PR China; ^2^ Department of Oncology, The Second Affiliated Hospital of Medical School of Xi'an Jiao Tong University, Xi'an, Shanxi Province, 710004, PR China

**Keywords:** nasopharyngeal carcinoma, radioresistance, signal transducer and activator of transcription 1(STAT1), lentivirus-mediated RNA interference

## Abstract

Radioresistance remains a major obstacle for clinicians in the treatment of nasopharyngeal carcinoma (NPC). Others and we have reported that signal transducer and activator of transcription 1 (STAT1) may be as an important gene for resistance to radiation. However, the relationship between STAT1 and radioresistance is still elusive. In this study, by constitutive silencing STAT1 in human radioresistant nasopharyngeal carcinoma CNE-2R cell line, we showed that inhibition of STAT1 enhanced radiosensitivity of CNE-2R. Furthermore, knockdown of STAT1 led to growth suppression and apoptosis promotion *in vitro* and *in vivo*. Moreover, cells with low STAT1 expression increased G2/M phase and decreased S phase at 2Gy. These result revealed that knockdown of stat1 expression could sensitizes the CNE-2R to radiotherapy, But the exact mechanism needs to be further clarified.

## INTRODUCTION

Nasopharyngeal Carcinoma is the most common cancer in Southeast Asia, especially in south China [1, 2]. Radiation has always been the major treatment method for nasopharyngeal carcinoma [3–5]. However, radiation therapy sometimes ineffective as cancer cells may be resistant to radiotherapy [6, 7]. Thus, it is essential to identify a novel target to reduce radioresistance and enhance the efficacy of radiotherapy for Nasopharyngeal Carcinoma.

Signal transducer and activator of transcription 1 (STAT1) has been identified to be associated with tumor radioresistance [8–10]. A variety of studies have shown that upregulation STAT1 expression is related to radio-resistance in many tumors, such as renal carcinoma cells, myeloma cell line, and breast cancer [9–12]. In addition, Hui et al. [13] reported that inhibition the expression of STAT1can improve radiosensitization in renal cell carcinoma cells.

In previous studies, our group found that STAT1 was over-expressed in radioresistant CNE-2R both at mRNA and protein level, and located at the key node of genetic interaction network, which also participated in many bioinformatics pathway [7, 14]. We speculated that STAT1 might contribute to radioresistance in CNE-2R cells. The currently study was designed to confirm whether STAT1 is correlated with radioresistance of CNE-2R *in vivo* and *in vitro*.

## RESULTS

### STAT1 knockdown in CNE-2R cells inhibited cell growth *in vitro* and *in vivo*

**Figure 1:**
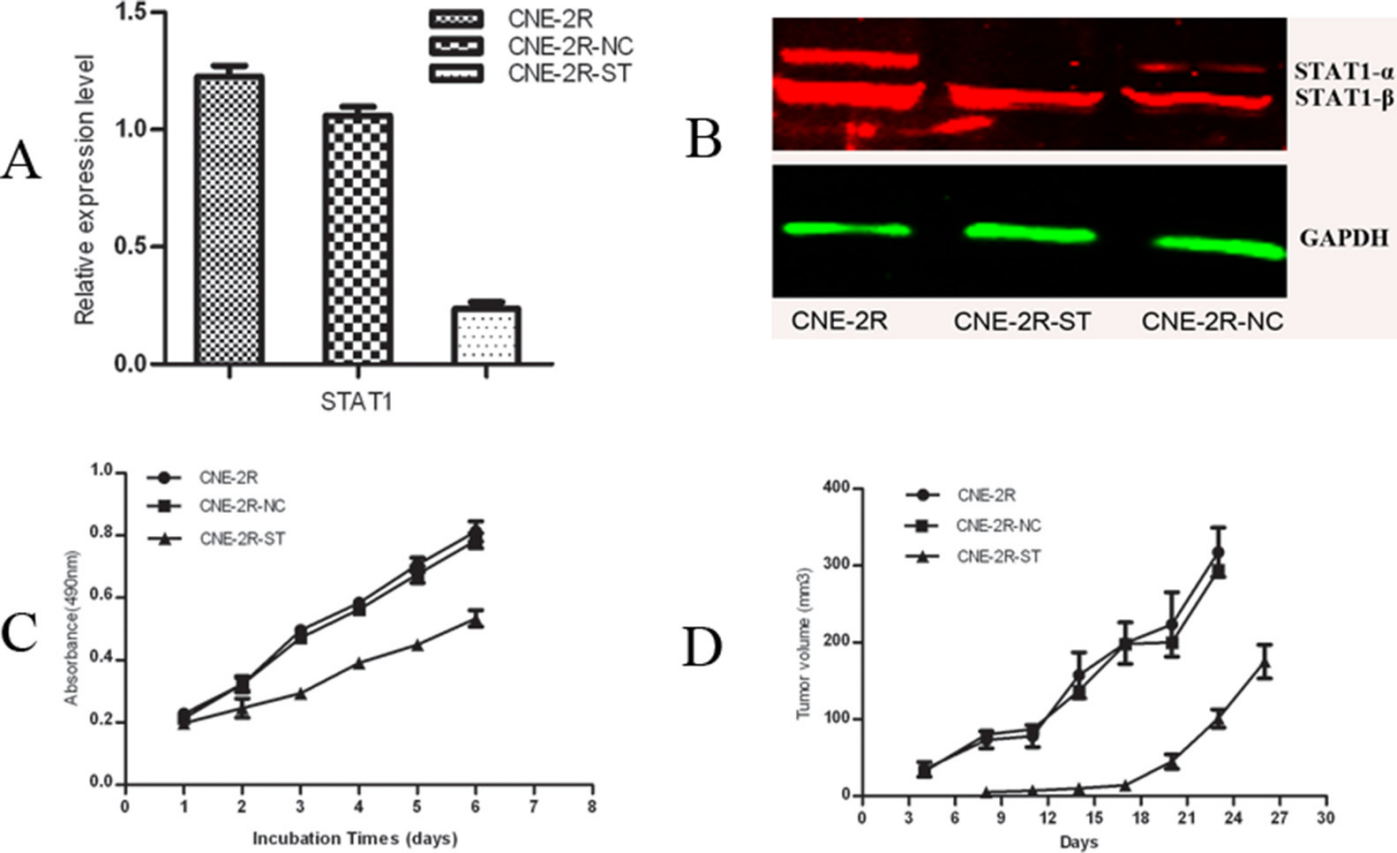
Inhibition of STAT1 decreases cell growth and tumor formation (**A**) The efficiency of STAT1 reduction was confirmed by quantitative real-time RT-PCR. The mRNA levels of STAT1 gene expression in CNE-2R, CNE-2R-NC and CNE-2R-ST cells were determined by RT-PCR. STAT1 expression was reduced by about 81% compared with the control. *P <* 0.05. (**B**) Detection of STAT1 protein expression by western blot. (**C**) STAT1 promotes CNE-2R cells growth. 3.0 × 10^3^ cells were seeded in each well of 96-well culture plates and relative cell number was measured everyday for 6 days. *P <* 0.05. (**D**) The tumor volume of each group. CNE-2R, CNE-2R-NC and CNE-2R-ST cells were injected into nude mice, tumor size was monitored every day and was calculated. Weight of the tumor was recorded at the end of the experiment. *P <* 0.05.

We knocked down the STAT1 expression in CNE-2R cells by RNAi technology. The efficiency of STAT1 reduction was confirmed by RT-PCR and Western Blot. As shown in Figure 1A, compared with CNE-2R and CNE-2R-NC cells, the level of STAT1 mRNA in CNE-2R-ST cells was decreased by about 81% (*p* < 0.05), while there were no significant difference between CNE-2R and CNE-2R-NC cells (*p* > 0.05). Figure 1B showed the STAT1 protein level reduced in CNE-2R-ST cells compared with CNE-2R and CNE-2R-NC cells, especially the level of STAT1-α protein. Growth curves were then constructed by cell counting. The results from MTT assays showed that cell growth rate of CNE-2R-ST was slower than CNE-2R-NC (*p* < 0.05), whereas no significant difference was found between CNE-2R and CNE-2R-NC (*p* > 0.05) (Figure 1C). Then we injected an equal number of cells into nude mice. We found that The relative tumor volume of the CNE-2R-ST mice was significantly smaller than that of the control groups (*P <* 0.05), whereas no significant difference was found between the CNE-2R-NC and CNE-2R (*P* **>** 0.05) (Figure 1D). As shown in Table 1, the tumor weight in the CNE-2R, CNE-2R-NC and CNE-2R-ST were 0.548 ± 0.08 g, 0.4738 ± 0.043 g, and 0.269 ± 0.035 g respectively. The results indicated that reduced STAT1 expression in CNE-2R inhibited tumor growth in nude mice.

**Table 1:** Tumor formation in nude mice

Cell types	Tumor weight (g)
CNE-2R	0.548 ± 0.08 g
CNE-2R-NC	0.4738 ± 0.043 g
CNE-2R-ST	0.269 ± 0.035 g

CNE-2R, CNE-2R-NC and CNE-2R-ST cells were injected into nude mice, Weight of the tumor was recorded at the end of the experiment.

### STAT1 knockdown in CNE-2R cells enhanced apoptosis *in vitro* and *in vivo*

**Figure 2:**
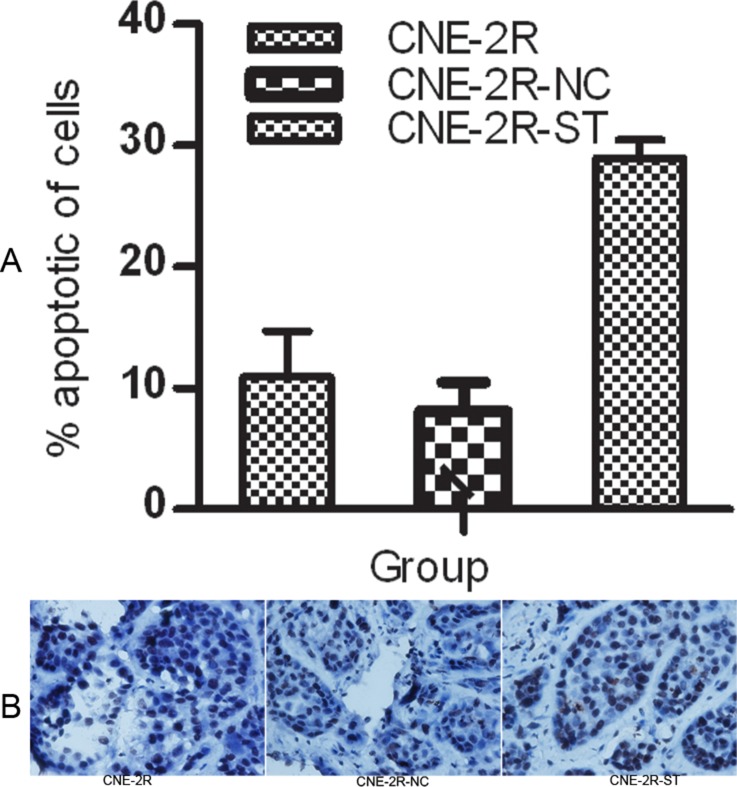
STAT1 inhibition might promote apoptosis (**A**) The rate of apoptosis was evaluated by flow cytometry. CNE-2R, CNE-2R-NC and CNE-2R-ST cells were cultured for 24 h, then stained with Anneix-V and 7-AAD following the instruction. (**B**) Tumor cells apoptosis was assessed by TUNEL method. The apoptosis rate of tumor cells in the CNE-2R-ST group was more than the CNE-2R-NC and CNE-2R group (original manification ×400), (*P <* 0.05).

We evaluated the effect of STAT1 knockdown on CNE-2R cells apoptosis *in vitro* and *in vivo*. *In vitro* experiment, the percentage of CNE-2R-ST cells apoptosis significantly increased to 28.97 ± 1.48%, while there were no significant differences in cell apoptosis between CNE-2R and CNE-2R-NC (10.95 ± 3.75%, 8.113 ± 2.38% respective, *P* **>** 0.05) (Figure 2A). In *vivo* experiment, the apoptosis rate of tumor cells in the CNE-2R-ST group was increased which was more than in the CNE-2R-NC and the CNE-2R group by TUNEL method (*P <* 0.05). (Figure 2B and Table 4)

**Table 2:** Related parameters of cell survival curve

Cell	SF2	α	β
CNE-2R	0.46	0.312 ± 0.043	0.037 ± 0.017
CNE-2R-NC	0.407	0.377 ± 0.068	0.034 ± 0.029
CNE-2R-ST	0.224	0.693 ± 0.036	0.024 ± 0.022

CNE-2R, CNE-2R-NC and CNE-2R-ST cells were seeded onto six-well plate and further irradiation with various doses (0–10 Gy). Cells were cultured for 10 day to allow colony formation. The dose–responses were analyzed using a linear-quadratic relationship model (LQ model): SF = e^–(αD+βD2 )^, where D is the single radiation dose and SF is the surviving fraction at dose D, Alpha and beta are radiobiological parameter.

**Table 3:** The cell cycle distribution was detected by flow cytometry

Dose (Gy)	Cell cycle phases (x ± SD, 100%)	CNE-2R	CNE-2R-NC	CNE-2R-ST
	G0/G1 (%)	56.22 ± 1.036	52.36 ± 1.006	57.27 ± 1.28
0 Gy	S (%)	22.73 ± 2.02	25.67 ± 0.96	25.23 ± 2.65
	G2/M (%)	21.05 ± 1.0	21.97 ± 1.95	17.50 ± 2.78
	G0/G1 (%)	55.23 ± 1.21	56.40 ± 0.92	55.45 ± 1.53
2 Gy	S (%)	19.43 ± 1.13	18.29 ± 1.39	9.40 ± 0.72^*^
	G2/M (%)	25.34 ± 0.85	25.31 ± 0.76	35.16 ± 0.8^*^

We observed the cell cycle distribution of three groups (CNE-2R, CNE-2R-NC and CNE-2R-ST cells) treated with 0 Gy irradiation, There was no obvious difference among these cells, we further studied the effect of STAT1 inhibition on cell cycle at 2 Gy, we found that the percentage of G2/M phase in the CNE-2R-ST cells increased after 2Gy radiation comparing with control cells, and the percentages of cells in S were decreased (*P <* 0.05).

**Table 4:** Evaluated the effect of STAT1 on CNE-2R cell apoptosis *in vivo*

Group	Cell apoptosis
CNE-2R	10.2 ± 2.1%
CNE-2R-NC	5.8 ± 0.6%
CNE-2R-ST	6.2 ± 0.3%

Tumor cell apoptosis was assessed by TUNEL method. We evaluated the effect of STAT1 on CNE-2R cell apoptosis *in vivo*. As shown in Table 4, the percentage of apoptotic tumor cells in CNE2R, CNE2R-NC and CNE2R-ST was 10.2 ± 2.1%, 5.8 ± 0.6%, 6.2 ± 0.3% by the terminal deoxynucleotidyl transferase-mediated dUTP-biotin nick end labeling (TUNEL) method (*P* < 0.05).

### Enhancing radiosensitivity of CNE-2R cells by RNA interference

**Figure 3:**
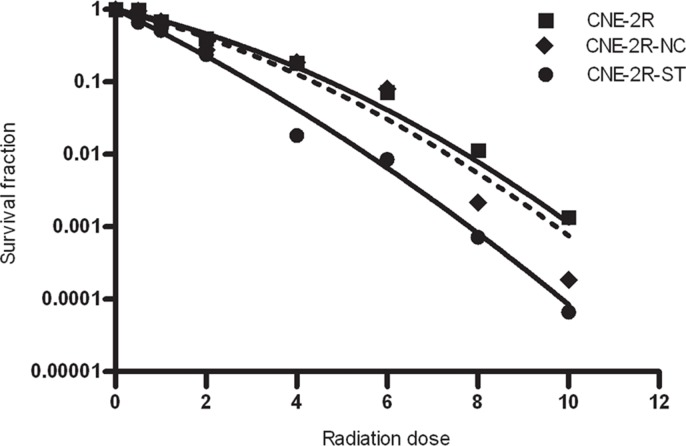
The effect of STAT1 inhibition on radiosensitivity CNE-2R, CNE-2R-NC and CNE-2R-ST cells were plated onto a six-well plate and were irradiated with X-rays at room temperature. The cells were exposed to doses of 0, 0.5, 1, 2, 4, 6, 8, 10 Gy. After irradiation, cells were cultured for 10 days in 5% CO2 atmosphere at 37°C.

To determine the effect of STAT1 expression on radiosensitivity of CNE-2R cells, colony formation assay was performed with CNE-2R, CNE-2R-NC and CNE-2R-ST cells. As were shown in Figure 3 and Table 2, CNE-2R-ST cells showed a significant decrease in SF2 which was 0.224, whereas CNE-2R-NC and CNE-2R cells, their SF2 were almost similar. The α/β ratios in CNE-2R, CNE-2R-NC and CNE-2R-ST were 8.432, 11.08, and 28.8 respectively. The results indicated that down-regulation STAT1 could improve radiosensitivity of CNE-2R cells.

### The changes of cell cycle induced by lentivirus-mediated RNAi

**Figure 4:**
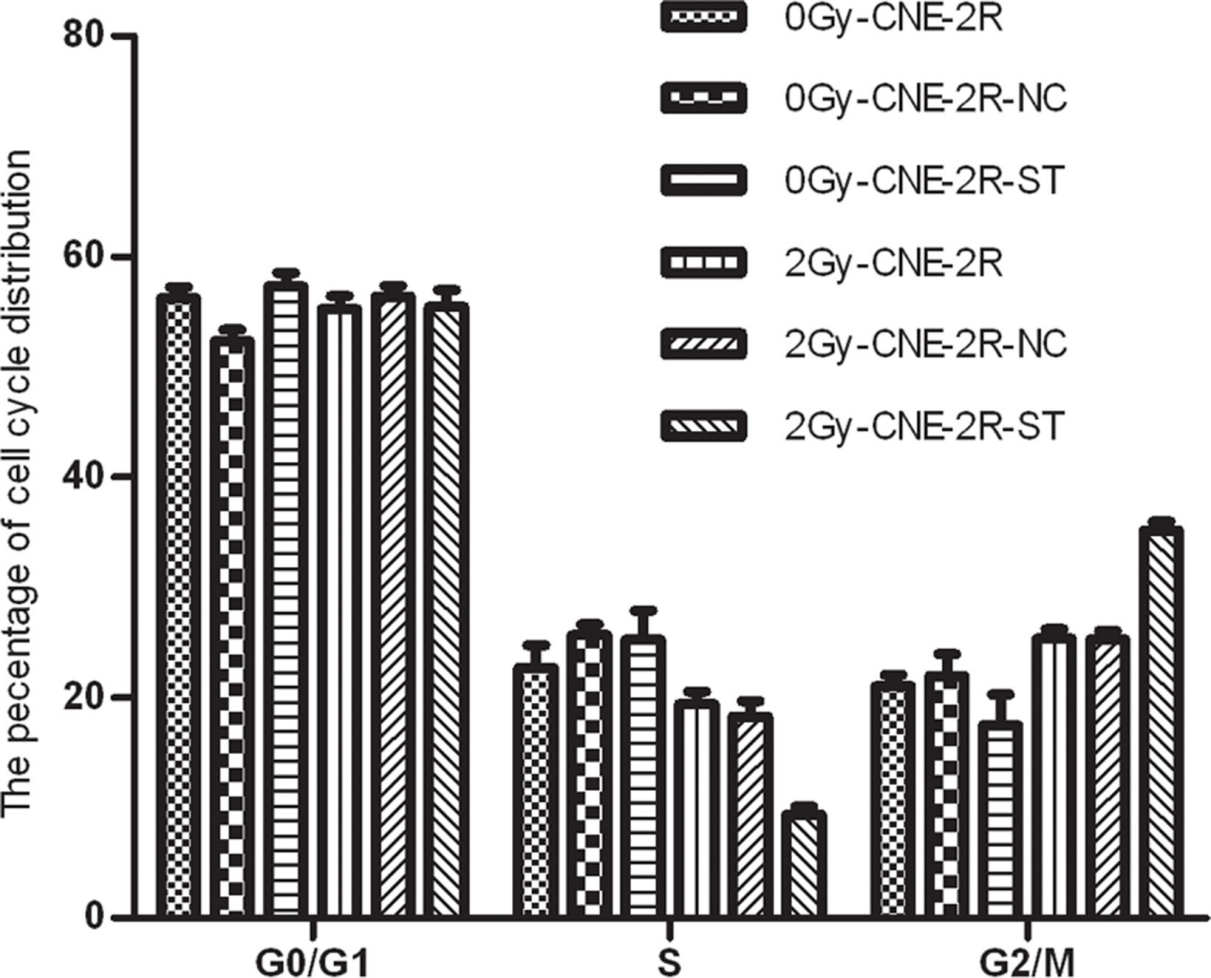
The effect of STAT1 inhibition on cell cycle The cell cycle distribution of these cells with or without STAT1 inhibitor was detected. The cell cycle distribution of these cells had no obvious difference after radiation at 0 Gy. However, the percentage of G2/M phase in CNE-2R-ST cells increased after radiation doses of 2 Gy comparing with the control cells. Meanwhile, the percentages of S phase in CNE-2R-ST cells decreased (*P <* 0.05).

To further examine the possible effect of STAT1 on cell cycle of CNE-2R cells, the cell cycle of the cells with or without STAT1 inhibitor was detected. In the absence of irradiation, the cell cycle distribution of these cells had no significant differences. However, upon 2 Gy radiation, a greater accumulation of CNE-2R-ST cells were found in the G2/M phase than those in the control cells, and the percentages of cells in S phase were decreased (*P <* 0.05) (Figure 4 and Table 3).

## DISCUSSION

Nasopharyngeal carcinoma (NPC) is a major malignant tumor of the head and neck region and is endemic in Southeast Asia, especially in Guangdong and Guangxi Province [15]. Radioresistance remains an important factor in relapse and metastasis for Nasopharyngeal carcinoma [4, 6]. Thus, it is imperative to understand the molecular mechanism of resistance and determine which genes prevent or interfere with resistance.

Signal transducer and activator of transcription 1 (STAT1) was up-regulated in CNE-2R, involved in many significant biological process, and associated with apoptosis and cell cycle genes [6]. Down-regulation of STAT1 expression might increase radiosensitivity of CNE-2R through inhibiting cell growth, improving apoptotic, and regulating cell cycle. In order to verify the hypothesis, we knocked down the expression of STAT1 in CNE-2R and explored the possible molecular mechanism between STAT1 and radio-resistance.

STAT1 expression has been reported to exhibit tumor suppressor. In renal cancer cells, down-regulation of STAT1 expression induced cell grew more slowly [9, 14]. Similar result has been obtained in our research. Pitroda et al. [11] revealed that knockdown of stat1 expression could sensitize the head and neck carcinoma cell to radiotherapy. Our data demonstrated that STAT1 inhibition induced radiosensitive *in vitro* (Figure 3 and Table 1). These results might indicate that STAT1 can improve CNE-2R cell growth, down-regulated STAT1 suppressed cell growth so as to sensitize the CNE-2R cell to radiation therapy.

We evaluated the effect of STAT1 on CNE-2R cell apoptosis *in vitro* and *in vivo*. As shown in Figure 2A, B, targeting of STAT1 could improve cell apoptosis. It indicated that the expression of STAT1 might suppress cell apoptosis. STAT1 has been reported to mediate pro-apoptotic or anti-apoptotic signals. It could also regulate apoptosis through a non-transcriptional mechanism by inhibiting the anti-apoptotic protein NF-kappaB [16]. In nu61 tumour, STAT1 might lead to radioresistance by impairing apoptotic [17]. Our data suggested that inhibition of STAT1 expression might increase apoptosis *in vitro* and *in vivo*. it means that STAT1 might restrain apoptosis. Our data was in agreement with previous study in that they both confirmed STAT1 may anti-apoptosis. We can infer that STAT1 inhibition induces radiosensitive by promoted apoptosis.

Previous study reported that regulation of cell cycle was crucial to radiation sensitivity. The cell cycle phase also determined a cell’s relative radiosensitivity, with cells being most radiosensitive in the G2/M phase, and least sensitive during the latter part of the S phase [18]. We then analyzed the cell cycle distribution of un-transfected and stably transfected CNE-2R cells. We found that down-regulation STAT1 expression induced the S phase was decreased and the G2/M phase was raised at 2 Gy radiation (Figure 4 and Table 3). Inhibition of STAT1 is convenient cells entering the G2/M phase which induce cells have not enough time to DNA repair [10]. We concluded that down-regulation of STAT1 expression lead to the S phase was declined and the G2/M phase was increased, which contributed to improve radiosensitivity of CNE-2R cells. STAT1 might participate in radioresistance through its function in the cell cycle.

In conclusion, STAT1 expression may be correlated with radioreisstance in CNE-2R cells. These result revealed that knockdown of stat1 expression could sensitizes the CNE-2R to radiotherapy. But the exact mechanism needs to be further clarified.

## MATERIALS AND METHODS

### Cell culture

We had successfully established CNE-2R cells in our previous research [7]. It was maintained in RPMI-1640 medium supplemented with 15% fetal calf serum. 293T cells was supplied by Cell Bank of Shanghai Institute of Cell Biology, The Cells were cultured in Dulbecco’s Modified Eagle’s Medium (DMEM). Both of them were cultivitated at 37°C in a humidified incubator with 5% CO2 atmosphere.

### Construction of STAT1 lentiviral vectors

Inverted and self-complementary hairpin DNA oligos targeting STAT1 mRNA and a negative oligonucleotide (NC) were designed and synthesized by Genchem Biotechnology Company (Shanghai, China). The sequences as follows: STAT1-shRNA, sense: 5′-CCGGCTGGAAGATTTACAAGATGAACTCGAG TTCATCTTGTAAATCTTCCAGTTTTTG-3′, Negative control shRNA, sense: 5′-CCGGTTCTCCGAAC GTGTCACGTTTCAAGAGAACGTGACACGTTCGGA GAATTTTTG-3′. All above sequences were synthesized, annealed, and ligated into linearized pGCSIL-GFP vector. The ligated DNA solution was transformed into E. coli DH5α, and incubated on a Luria Bertani (LB) plate (LB solid medium containing 50 ng/l ampicillin and 2% agarose gel) at 37°C for 16 h. Positive clones were identified by DNA sequence analysis (Majorbio Biotech Co., Ltd., Shanghai, China). Lentiviral vector DNAs and packaging vectors were then transfected into 293T cells. Supernatants containing lentiviruses were harvested 48 h later after transfection. Then, we performed subsequent purification using ultracentrifugation and the titer of lentiviruses was determined.

### Transfection of lentivirus

CNE-2R was plated in antibiotic-free 1640 at the density of 1 × 10^5^ /well in the 6-wells, when the cell grew to approximately 70–80% confluence of flask, CNE-2R was transduced with the established lentivirus vector (Multiply of infection, MOI = 10). After 48 h, we observed green fluorescence by Inverted fluorescence microscope. For improving transfection efficiency, the transfected CNE-2R cells was selected by fluorescence activated sorter. CNE-2R cells transfected with lentivirus-mediated shRNA targeted STAT1 was named CNE-2R-ST, transfected with lentivirus-mediated shRNA (NC) was named CNE-2R-NC.

### Real-time RT-PCR

Total RNA was extracted from CNE-2R using Trizol reagent (Invitrogen, cat: 15596026). Reverse-transcribed with MLV reverse transcriptase (Invitrogen, cat: 18080044) and random primers. STAT1 primer was designed and synthesis, the length of STAT1 product was 171bp, sense: CCAAAGGAAGCACCAGAGCC; antisense: AGAGCCCACTATCCGAGACACC. Quantitative PCR amplification was done with a 20 μl reaction mixture, consisting of 2 μl reverse transcription reaction mixture, 0.8 μl sense and antisense primers and 9 μl PCR master mixture, 7.4 μl double steaming water to 20 μl. The PCR conditions were as follows: initial denaturation at 94°C for 3 min; 32 cycles of denaturation at 94°C for 30 s; annealing at 55°C for 30 s; elongation at 72°C for 30 s, and final extension at 75°C for 5 min. The STAT1 expression was normalized using the expression of GAPDH, The *p*-values was calculated based on a Student’s *t*-test of the replicate 2(-Delta Ct) values for each gene in the control group and experiment group, and *p* values less than 0.05 were considered statistically significant.

### Western blot analysis

Cells were washed with ice-cold PBS and lysed at 4°C. The lysaten was centrifuged with 12,000 rpm at 4°C for 30 min. The BCA Protein Assay kit (Beyotime, Nanjing, China) was used to determine the protein content in the supernatants. About 30 μg of protein was separated on 10% SDS-PAGE and transferred to polyvinylidene difluoride (PVDF) membranes (Solarbio, PeKing). After being blocked with 5% nonfat milk for 1 h, the membrane was incubated with different antibodies required at 4°C overnight, followed by adding corresponding secondary antibody at room temperature approximately 2 h. The signal was then detected and quantified with Odyssey infrared imaging system (LI-COR Biosciences, Lincoln, NE, USA). STAT1 antibody was obtained from Cell Signaing Technology (9172, Massachusetts, US). The GAPDH primary antibody was purchased from Boster Co. (BA2913, Wuhan, China), and the goat anti-mouse/rabbit IgG secondary antibody was purchased from the KPL Co.

### Measurement of cell proliferation by methylthiazoletetrazolium assay

The cell viability was measured by MTT assay. 3.0 × 10^3^ cells were plated into each well of 96-well culture plates, and relative cell number was measured everyday for 6 days. Each group set up three parallel plates. After incubated 24 h, the medium was aspirated and 20 μl of MTT (5 mg/ml) was added to the cells. After 4 h incubation, the media were aspirated and 150 μL of dimethyl sulf-oxide was added. Shaking the plates at room temperature for 10 min, Optical densities were determined on a Versamax microplate reader (Thermo, USA) at a wavelength of 490 nm.

### Detection apoptosis analysis by flow cytometry

A total of 1.0 × 10^5^ mock or stably transfected CNE-2R cells were seeded in the 6-wells and were cultured for 24 h. Cells were harvested by trypsinization, washed twice with PBS, re-suspended in 500 μl PBS, stained with Anneix-V and 7-AAD following the instruction (Becton Dickinson, cat: BD 559763), and then immediately analyzed by flow cytometry. The experiments were performed in triplicate.

### *In vitro* colony formation assay

For cloning assay, cells were plated onto a six-well plate and were irradiated with X-rays at room temperature. The cells were exposed to doses of 0, 0.5, 1, 2, 4, 6, 8, 10 Gy. After irradiation, cells were cultured for 10 days in 5% CO2 atmosphere at 37°C. The colonies were fixed with carbinol and stained with 0.1% Giemsa (Solarbio, cat:51811-82-6). Colonies containing more than 50 cells were scored as sur*vivo*rs. All experiments were performed three times. The survival fraction was calculated as the experimental group numbers of colonies divided by the numbers of cells seeded times plating efficiency. Plating efficiency was calculated as the control group numbers of colonies divided the numbers of cells seeded times 100%. The dose–responses were analyzed using a linear-quadratic relationship model (LQ model): SF = e^–(αD+βD2)^, where D was the single radiation dose and SF was the surviving fraction at dose D, Alpha and beta were radiobiological parameter. Graphpad Prism 4 software was used to create fit cure.

### Cell cycle assay

Cells were harvested 24 h after treatment, washed in cold sterile phosphate-buffered saline and ixed with 70% ethanol at 4°C over night. Cells were washed twice with PBS, re-suspended in 500 μl PBS, stained with propidium iodide, and then immediately analyzed by flow cytometry.

### Xenograft tumors in nude mice

Equal numbers of 1.0 × 10^7^ untransfected or stably transfected CNE-2R cells were harvested and subcutaneously injected into 4 to 6 week old BALB/c nude mice (Department of Laboratory Animal, Guang Xi Medical University) which were maintained under pathogen-free conditions. The tumors volume was calculated by the following formula: V = 0.5 × A × B^2^, where A was the length and B was the width of tumor. At 30 days after inoculation, all mice were sacrificed and the weights of tumors were measured at the end of the experiment. For all the experiments, animal handling and experimental procedures were approved by the Animal Experimental Ethics Committee of Guangxi Medical University.

### Analyses of xenograft tumor cell apoptosis by TUNEL assay

A TUNEL assay was performed for all groups according to the manufacturer’s protocol (ApopTag S7100; Chemicon International, Billerica, MA). Counting apoptotic cells in 10 arbitrarily selected fields at ×400 magnification under optical microscope (Olympus, Japan). The apoptotic rate (per ×400 microscopic fields) was calculated as number of apoptotic cells ×100/total number of cells.

### Statistical analysis

All experiments were performed at least three times. Statistical analysis was performed using SPSS 17.0 software. Data were expressed as the means of three different experiment ± SD. The Student’s *t*-test was used to evaluate the significant difference of two groups of data in all the experiment. *P* < 0.05 was thought to be significantly different for two groups.
